# Integrin-FAK signaling rapidly and potently promotes mitochondrial function through STAT3

**DOI:** 10.1186/s12964-016-0157-7

**Published:** 2016-12-15

**Authors:** Nishant P. Visavadiya, Matthew P. Keasey, Vladislav Razskazovskiy, Kalpita Banerjee, Cuihong Jia, Chiharu Lovins, Gary L. Wright, Theo Hagg

**Affiliations:** Department of Biomedical Sciences, Quillen College of Medicine, East Tennessee State University, Building 178, Maple Ave, PO Box 70582, Johnson City, TN37614 USA

**Keywords:** Bioenergetics, Cell death, CRISPR, ECM, Endothelial cell, Focal adhesion kinase, Integrin, Mitochondria, Vitronectin, STAT3

## Abstract

**Background:**

STAT3 is increasingly becoming known for its non-transcriptional regulation of mitochondrial bioenergetic function upon activation of its S727 residue (S727-STAT3). Lengthy mitochondrial dysfunction can lead to cell death. We tested whether an integrin-FAK-STAT3 signaling pathway we recently discovered regulates mitochondrial function and cell survival, and treatments thereof.

**Methods:**

Cultured mouse brain bEnd5 endothelial cells were treated with integrin, FAK or STAT3 inhibitors, FAK siRNA, as well as integrin and STAT3 activators. STAT3 null cells were transfected with mutant STAT3 plasmids. Outcome measures included oxygen consumption rate for mitochondrial bioenergetics, Western blotting for protein phosphorylation, mitochondrial membrane potential for mitochondrial integrity, ROS production, and cell counts.

**Results:**

Vitronectin-dependent mitochondrial basal respiration, ATP production, and maximum reserve and respiratory capacities were suppressed within 4 h by RGD and αvβ3 integrin antagonist peptides. Conversely, integrin ligands vitronectin, laminin and fibronectin stimulated mitochondrial function. Pharmacological inhibition of FAK completely abolished mitochondrial function within 4 h while FAK siRNA treatments confirmed the specificity of FAK signaling. WT, but not S727A functionally dead mutant STAT3, rescued bioenergetics in cells made null for STAT3 using CRISPR-Cas9. STAT3 inhibition with stattic in whole cells rapidly reduced mitochondrial function and mitochondrial pS727-STAT3. Stattic treatment of isolated mitochondria did not reduce pS727 whereas more was detected upon phosphatase inhibition. This suggests that S727-STAT3 is activated in the cytoplasm and is short-lived upon translocation to the mitochondria. FAK inhibition reduced pS727-STAT3 within mitochondria and reduced mitochondrial function in a non-transcriptional manner, as shown by co-treatment with actinomycin. Treatment with the small molecule bryostatin-1 or hepatocyte growth factor (HGF), which indirectly activate S727-STAT3, preserved mitochondrial function during FAK inhibition, but failed in the presence of the STAT3 inhibitor. FAK inhibition induced loss of mitochondrial membrane potential, which was counteracted by bryostatin, and increased superoxide and hydrogen peroxide production. Bryostatin and HGF reduced the substantial cell death caused by FAK inhibition over a 24 h period.

**Conclusion:**

These data suggest that extracellular matrix molecules promote STAT3-dependent mitochondrial function and cell survival through integrin-FAK signaling. We furthermore show a new treatment strategy for cell survival using S727-STAT3 activators.

## Background

Integrins are heterodimer transmembrane receptors which bind ECM molecules to promote cell adhesion and initiate intracellular signaling that can lead to cell survival [1, 2]. Disruption of integrin binding can cause cell death, especially for cells attached to basement membranes [3], e.g., endothelial cells in the central nervous system (CNS). Among others, endothelial cells express αvβ3 integrins which contribute to their survival [4, 5]. Integrin signaling is important for normal endothelial cell function in maintaining the blood-brain-barrier (BBB) [6, 7], whose disruption by neural injury and stroke leads to disease progression [8]. FAK is one of the major integrin signaling mediators and is activated via autophosphorylation on Y397 [9] which can suppress apoptosis in endothelial cells [10].

Mitochondria not only play a vital role in energy production, particularly in the CNS [11], but also have emerged as a key stress-signaling hub within the cell [12]. CNS endothelial cells have a very high mitochondrial mass compared to those of other organs [13], and mitochondrial function is important for maintaining the BBB and ATP-dependent trans-endothelial transport [13, 14]. Mitochondrial dysfunction after neurological insults plays a role in BBB breakdown and tissue degeneration [7, 15, 16]. Lengthy mitochondrial bioenergetic dysfunction leads to depletion of ATP, increased production of reactive oxygen/nitrogen species, calcium dysregulation, and release of pro-apoptotic proteins, leading to cell death [17, 18].

Integrins can prevent apoptosis through FAK-AKT signaling [10, 19, 20], and inhibiting mitochondria-associated bit1 [20, 21], but have not been implicated in bioenergetic function. We recently discovered an integrin signaling pathway that inhibits CNTF expression, involving FAK, JNK and the S727 residue of the transcription factor STAT3 [22]. Depending on phosphorylation of residues S727 or Y705, STAT3 can inhibit or promote nuclear gene expression [23]. Recent seminal studies identified a non-transcriptional role of pS727-STAT3 in stimulating mitochondrial bioenergetic function through electron transport chain (ETC) complex I, II and V activity [24–26], probably not by binding directly [27], but by binding to prohibitin 1 [28]. STAT3 can also reduce formation of the mitochondrial permeability transition pore, possibly by interacting with cyclophilin D [29], thus maintaining membrane potential necessary for bioenergetic function, as well as preventing release of cytochrome-c, which leads to apoptosis [30]. STAT3 can also reduce ROS formation, possibly by improving complex I coupling, improving cell survival under ischemic conditions [30, 31]. It is unknown whether integrin or FAK signaling interacts with pS727-STAT3 to regulate mitochondrial function.

A few studies have identified mechanisms that activate S727-STAT3, including cytoplasmic PKC isoforms [32, 33] and the c-Met receptor acting via ERK and AKT [34, 35]. The small molecule bryostatin 1 activates PKC [36] whereas HGF activates c-Met [34]. They are being developed for treating Alzheimer’s disease [37] and spinal cord injury [38], respectively. Stimulating S727-STAT3 more directly may be a treatment strategy that can circumvent pathologically disrupted integrin or FAK signaling.

The present study determined whether ECM-integrin-FAK-STAT3 signaling promotes mitochondrial function in cultured endothelial cells and whether treatments that activate STAT3 could rescue cells against the loss of FAK signaling.

## Methods

### Cell culture

The immortalized mouse brain endothelioma cell line (bEnd5) was created and characterized as a good model for CNS endothelial cells [39–41]. Culture medium components were from Gibco. The cells were grown in in T-75 Flask (Cat # CC7682-4175, CytoOne, Ocala, FL) in DMEM supplemented with 10% fetal calf serum, 3 mM glutamine, 100 units/ml penicillin and 100 μg/ml streptomycin, 1 mM sodium pyruvate, 1% non-essential amino acids. Cells were passaged every seven days with cells up to 35 passages being used for experiments. Before use, replated cells were maintained for 24 h except where noted specifically. The cells were counted in high resolution images obtained with a 10× objective, five fields per well, using ImageJ software (NIH), with 3–5 wells per condition.

### Mitochondrial bioenergetics measurements

Mitochondrial bioenergetics measurements were made with an XF24 Extracellular Flux Analyzer (Seahorse Bioscience, North Billerica, MA). Cell density was optimized for recommended basal OCR ranges of 50–400 pMole/Min. For vitronectin (VTN) experiments, XF-24 culture plates were incubated with poly-d-lysine for 1 h (50 μg/ml; Sigma), washed 3× with autoclaved H_2_O, dried and coated with recombinant human VTN (0.5 μg/cm^2^, Cat#SRP3186, Sigma) for 1 h. BEnd5 cells were seeded at 100,000/well with or without RGDS (Arg-Gly-Asp-Ser peptide, 100 μg/ml, Cat#3498, Tocris, Bristol, UK) or P11 (His-Ser-Asp-Val-His-Lys-NH_2_ peptide, 10 μg/ml, Cat#4744, Tocris) peptides in serum-free medium supplemented with 2% B27 (Gibco cat# 10889038) for 4 h. We also tested the effects of VTN, EHS mouse laminin-111 (Cat#L2020, Sigma) and human plasma fibronectin (Cat#F3879, Sigma) as 50 μg/ml substrate with 10 μg/ml added to serum-free medium for 4 h. For the other experiments, 50,000 bEnd5 cells/well were seeded without substrate and grown for 44 h before 4 h incubation with FAK14 (5 or 10 μM, Cat#3414, Tocris) or stattic (5 or 10 μM, Cat#2798, Tocris) with or without bryostatin (50 nM, Cat#2383, Tocris) or HGF (500 ng/ml, Cat#315-23, PeproTech, Rocky Hill, NJ) or actinomycin D (0.3 μg/ml, Cat#1229, Tocris) treatment with or without 5 μM FAK14. After treatments, cells were washed once with XF base media supplemented with 2.5 mM glucose and 1 mM sodium pyruvate, and incubated for 1 h in a non-CO_2_ incubator. Before use, sensor cartridges were hydrated, loaded with oligomycin (Cat# 495455, Millipore), carbonyl cyanide 4-(trifluoromethoxy) phenylhydrazone (FCCP, Cat# 0453, Tocris, 1 μM as used by others [42, 43], antimycin A (Cat# A8654, Sigma), and calibrated according to manufacturer’s instructions. XF24 plates with cells were then loaded and mitochondrial respiration measurements performed according to a standard software protocol. The basal OCR, ATP production, maximum reserve and respiratory capacity were calculated as described [43], with averages calculated from five wells per condition in each individual experiment. Bioenergetics data are presented as a representative original trace (OCR raw data) while bar graphs were normalized to the average of controls for a set of experiments.

### CRISPR-Cas9 mediated knockdown of STAT3

CRISPR-Cas9 pre-cloned guide RNA cassettes targeting mouse STAT3 were purchased from Origene (Cat# KN316845) and knockdown performed according to [44] with some alterations. Briefly, 2 × 10^5^ bEnd5 cells were seeded into 6-well plates and maintained for 24 h. Lipofectamine 3000 (Cat# L3000, Invitrogen) was used at 0.5% complexed with 5 μg plasmid (2.5 μg Cas9-gRNA with 2.5 μg Donor) in Opti-MEM. Transfections were performed according to standard procedures, with cells maintained for 24 h after transfection. Transfection medium was removed from cells the following day and replaced with fresh medium containing 2 μg/ml puromycin (Cat# A1113803) which caused 100% cell death in non-transfected control cells after 4 days. Cells resistant to puromycin treatment were grown in fresh medium and maintained until confluent (~7–14 days). PCR primers were designed against regions of the mouse STAT3 gene flanking the guide-RNA target sequence and were: Forward 5′-GGCCTTGACCTGTCTGTCAT -3′ and reverse 5′-TGTGCAGAGATCTCACCAAGT -3′ to generate an amplicon of 849 bp in a reaction with 100 ng of genomic DNA extracted from cells with the DNeasy Blood and Tissue kit (Qiagenssa, USA, Cat# 69506). PCR products were run on an agarose gel for purification and sequencing performed by the Molecular Core Facility at ETSU using the following primer 5′-GGCCTTGACCTGTCTGTCAT -3′. A point mutation was identified at the predicted Cas9-guide RNA cut site, resulting in a frame shift. Knockout of protein was confirmed by western blots.

### Subcellular fractionation

Mitochondria were isolated as previously described [45]. Briefly, cells were homogenized in ice-cold isolation buffer (215 mM mannitol, 75 mM sucrose, 0.1% bovine serum albumin, 20 mM HEPES, 1 mM EGTA; pH adjusted to 7.2 with KOH), followed by centrifugation, twice at 1,300 g for 3 min to obtain nuclear pellets. The supernatant was centrifuged at 13,000 g for 10 min to obtain mitochondrial pellets. The last supernatant was collected as the cytosolic fraction. The cold and the EGTA in the isolation buffer ensure that proteins remain phosphorylated and intact. Moreover, we have found that addition of protease and phosphatase inhibitors (Cat# P8340, Sigma), supplemented with 1 mM sodium orthovanadate, does not reveal any differences. The purity of sub-cellular fractions was confirmed using Western blotting for fraction-specific markers. For another experiment, mitochondria were treated with stattic (10 μM) or okadaic acid (1 μM; Cat# 5934, Cell Signaling) for 2 h in KCl-based respiration buffer (125 mM KCl, 2 mM MgCl_2_, 2.5 mM KH_2_PO_4_, 20 mM HEPES and 0.1% bovine serum albumin, pH 7.2) containing oxidative substrates pyruvate (5 mM) and malate (2.5 mM) before protein extraction.

### Western blot analysis

Protein from whole cells was extracted using 1% RIPA lysis buffer (cat# R0278, Sigma) with standard protease and phosphatase inhibitors (Cat# P8340, Sigma). Proteins from subcellular fractions and whole cell lysate were separated by SDS–PAGE using Criterion 4–20% Tris–HCl (10–250 kD) gels (Bio-Rad, Hercules, CA). After transfer to PVDF membranes, and after washing in TBST buffer containing 0.1% Tween 20, and blocking in 5% milk in TBST, the primary antibodies were diluted in 5% milk in TBST and incubated at 4 °C for 18 h. Primary antibodies against pyruvate dehydrogenase (PDH, 1:2,500, Cat#3205), pS727-STAT3 (1:400, Cat#9134), STAT3 (1:1,000, Cat#12640), pY397-FAK (1:500, Cat#3283), FAK (1:1,000, Cat#3285), α-tubulin (1:2,000, Cat#2125) all from Cell Signaling Technology (Danvers, MA) and histone 3H3 (1:15,000, ab1791) and ATPase (1:5,000, ab14730) from Abcam (Cambridge, MA). After washing in TBST, the membranes were incubated in species- and isotype-specific HRP-conjugated secondary antibodies in TBST (1:2000, Cat#7074, Cat#7076, Cell Signaling). Chemiluminescence (ECL, Cat# 34080, ThermoFisher) was used to reveal immunoreactive protein bands, detected by X-ray film or imaged on a Licor Odyssey FC (Lincoln, NE) for 10 min.

### Quantitative capillary ProteinSimple westerns

For one of the FAK inhibition experiments, quantitative analysis of protein expression was performed according to the ProteinSimple protocol guide with reagents of a kit (Cat#, SM-W004 and DM-001, ProteinSimple), except where noted. Briefly, cell lysates were diluted to 0.2 μg/μl with 0.1× sample buffer supplemented with 1× fluorescent molecular weight markers and 40 mM DTT for a 5 μl reaction (1 μg protein/reaction). Samples were heated at 95 °C for 5 min before loading into 24 single designated wells of a pre-filled plate along with blocking reagent, primary antibodies (1:25, pS727-STAT3, 1:50, tSTAT3, or 1:500, α-tubulin and ATPase) in antibody diluent, with pSTAT3 and tubulin/ATPase or STAT3 and tubulin/ATPase mixed together for detection within the same capillary), anti-rabbit HRP conjugated secondary antibody, luminol-peroxide mix to generate chemiluminescence and washing buffer. Plates were loaded into the automated ProteinSimple “Wes” for electrophoresis and fluorescence imaged in real time by a CCD camera for immunodetection in the capillary system at default settings: Electrophoresis, 375 volts, 25 min; blocking, 5 min; primary antibody, 30 min; secondary antibody, 30 min. Data was analyzed using Compass software (ProteinSimple) and expressed as peak intensity or synthetic bands. Quantification was performed by normalizing areas under protein peaks to α-tubulin loading control.

### siRNA and STAT3 plasmid transfections

SiRNAs against mouse FAK (Cat# L-041099, Dharmacon) or a non-targeting negative control (Cat# D-001810-10, Dharmacon) were transfected into bEnd5 cells using lipofectamine-2000 (Cat #11668, Invitrogen) 24 h after plating cells in Seahorse plates at 12,500 cells/well, with 50 nM siRNA and 0.5% Lipofectamine according to manufacturer’s protocol. A second transfection was performed 24 h later (48 h after plating) due to FAK’s long protein half-life [46] and bioenergetics assessed 5 days later. Transfection efficiency was >80% as visualized by fluorescent microscopy using siGLO RNA (Cat #D-001630-02, Dharmacon). Mutant S727A STAT3 expression plasmid was obtained from Addgene (Cat# 8708) while wild type mouse STAT3 was PCR amplified from cDNA and directionally cloned into a pcDNA 3.1(+) expression vector using BamHI and EcoRI restriction sites. bEnd5 cells were transfected with 500 ng plasmid per well using lipofectamine 3000 (Cat# L3000, Invitrogen) and maintained for 24 h prior to Sea Horse analysis.

### TMRM measurements

For confocal imaging, bEnd5 cells were seeded at 10^5^ cells/35 mm clear bottom culture dishes (P35G-1.5–14-c, MarTek Corp, Ashland, MA). After 24 h, cells were treated with FAK14 (5 or 10 μM) for 4 h, followed by 30 min incubation with 20 nM tetramethylrhodamine methyl and ethylesters, which accumulate within active mitochondria in a potentiometric fashion (TMRM, Cat#T668, Invitrogen) and 20 μM Hoechst 33342 (Cat # 62249, ThermoFisher Scientific) dyes. The cells were imaged for TMRM red fluorescence (544/590 nm) and nuclear Hoechst blue fluorescence (460/490 nm) using a Leica SP8 confocal microscope.

For spectrofluorometry, bEnd5 cells were grown for 24 h at 10,000/well in 96 wells (Cat#3997, Corning) and treated with FAK14 for 4 h. Afterwards, mitochondrial membrane potential was estimated by incubation with 25 nM TMRM and 300 nM DAPI (Dilactate, Invitrogen), to normalize for the number of cells, for 30 min at 37 °C. Fluorescence (TMRM: 544/590, DAPI 360/450 nm excitation/emission) was measured with a plate reader. O_2_^•¯^ formation was measured by incubation in 10 μM 2-,7-dichlorodihydrofluorescein diacetate (DCFH_2_-DA, Cat# D6883, Sigma) and 5 U/ml horseradish peroxidase (HRP, CatP8375, Sigma) as described before [45]. Oxidized DCF fluorescence (485/520 nm) was measured after 10 min at 37 °C. H_2_O_2_ production was measured using 1 μM Amplex Red (Cat#A36006, Invitrogen) and 0.25 U/ml HRP at 30 °C as described [45]. Formation of fluorescent resorufin from Amplex Red was measured (530/590 nm).

### Statistical analyses

Statistical analyses were performed with Students t-test for two samples or with a One Way Analysis of Variance (ANOVA) followed by a Bonferroni post-hoc test using GraphPad Software (La Jolla, CA). The number of experiments indicated in the text are independent experiments.

## Results

### Integrin antagonists inhibit mitochondrial function

**Fig. 1:**
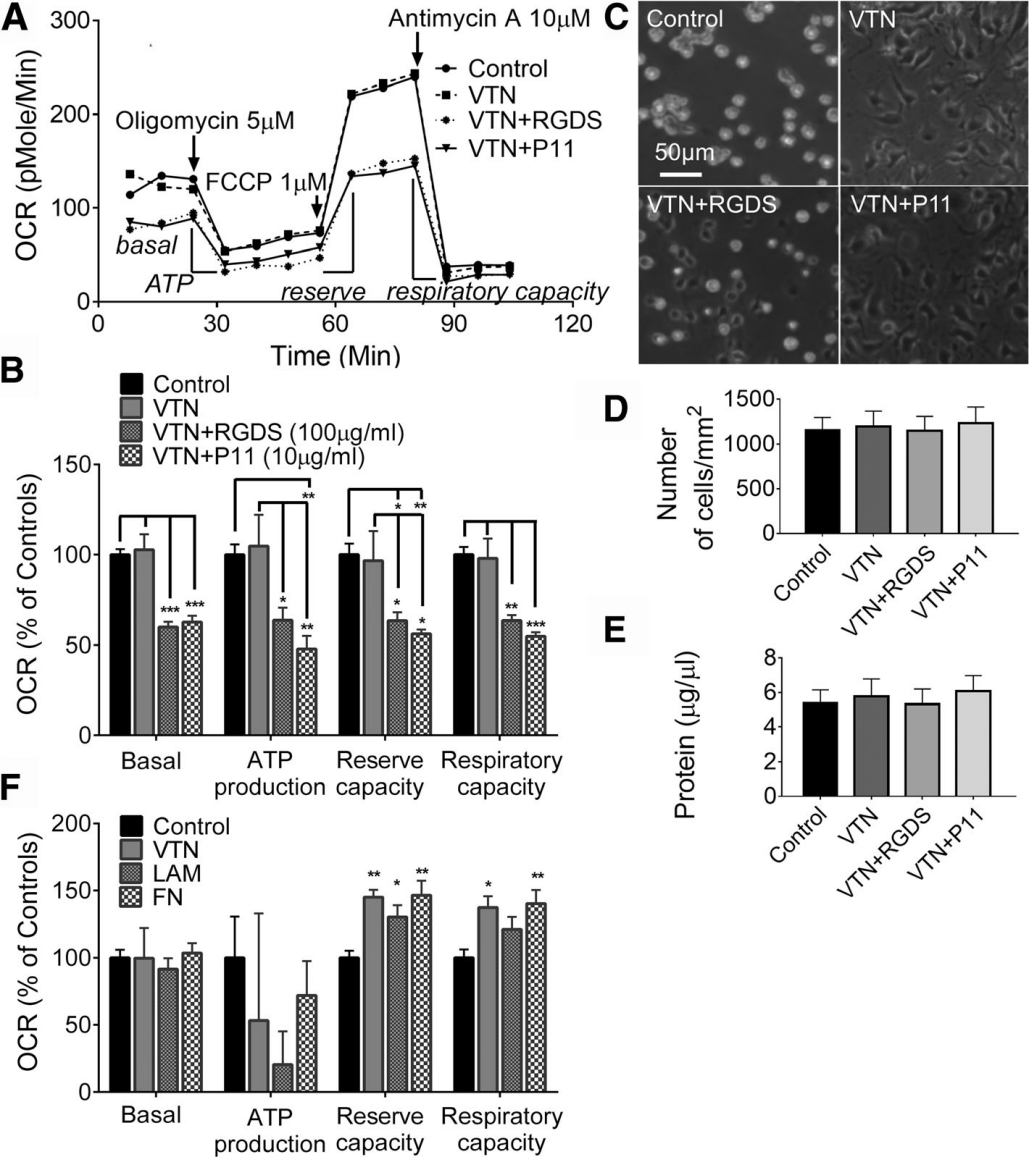
RGD and αvβ3 integrin blockade suppresses mitochondrial bioenergetics. **a** Traces from a representative experiment show mitochondrial bioenergetic deficits in mouse brain bEnd5 cells plated on vitronectin (VTN), in the presence of RGD integrin (RGDS) or αvβ3 integrin (P11) blocking peptides, in serum free medium for 4 h, as measured by oxygen consumption rate (OCR) in an XF24 Seahorse Flux Analyzer. Data are means of 5 wells per condition. **b** Integrin blockade reduced all four measures of cellular respiration relative to control (no VTN) and VTN treated cells. **c** Cells cultured in XF24 microplates for 4 h on no substrate or on VTN, with or without integrin blockade, seemed similar in number but cells without substrate and in the presence of RGDS were more rounded. The cell number (**d**) and protein content (**e**) in such cells, determined without subsequent Seahorse analyses, were not significantly different across all conditions. Cell counts were independently verified. **f** Cells cultured for 4 h with VTN, laminin (LAM) or fibronectin (FN) as substrate and added to serum-free media showed increased mitochondrial bioenergetics. All data are means ± SEM of 3–4 independent experiments. *p < 0.05, **p < 0.01, and ***p < 0.001

BEnd5 endothelial cells were cultured for 4 h in serum-free conditions on VTN substrate to provide a ligand for integrins, especially αvβ3 integrin present on endothelial cells. The tetrapeptide RGDS, which blocks RGD binding ligands [47], as well as the P11 peptide which blocks αvβ3 integrin [48], substantially inhibited mitochondrial function (Fig. 1a), with significantly reduced ATP production, maximum reserve capacity and maximum respiratory capacity (Fig. 1b). The 4 h incubation with RGDS and P11 did not cause any obvious changes in cell number (Fig. 1c, d), or protein content (Fig. 1e), indicating that the effects were not due to cell detachment. Conversely, maximum reserve and respiratory capacity were stimulated when integrin agonists VTN, laminin or fibronectin were added directly to bEnd5 cell culture medium for 4 h under serum-free conditions (Fig. 1f).

### FAK inhibition causes mitochondrial dysfunction

**Fig. 2:**
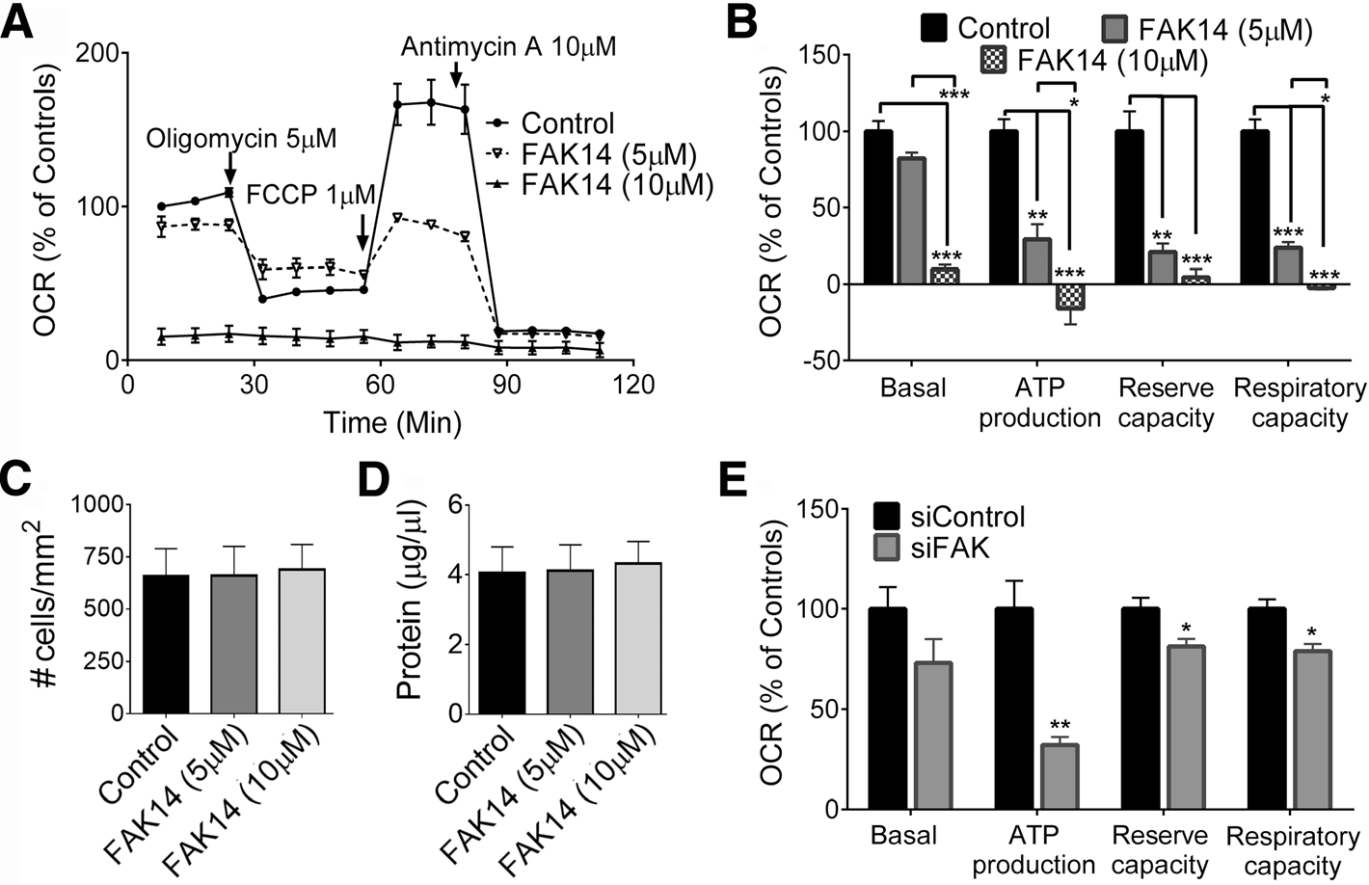
FAK inhibition causes mitochondrial dysfunction. **a** Pharmacological inhibition of the integrin signaling molecule, FAK, with FAK14 treatment of bEnd5 cells for 4 h produced a robust and dose-dependent suppression of mitochondrial bioenergetic function as shown in a representative OCR trace. Cells were grown for 44 h without substrate before treatment. **b** FAK14 causes reductions in basal respiration, ATP production, and maximum reserve and respiratory capacity. The cell number (**c**) and protein content (**d**) were not different across conditions. Cell counts were independently verified. **e** SiRNA-mediated knockdown of FAK (siFAK) decreased ATP production, reserve capacity and respiratory capacity relative to a non-targeting siRNA control (siControl). Data are mean ± SEM from 3 independent experiments. *p < 0.05, **p < 0.01, ***p < 0.001, ns = not significant versus control vehicle group

FAK inhibitor 14 (FAK14, also named Y15: 1,2,4,5-Benzenetetramine tetrahydrochloride, MW = 284) is a water-soluble small-molecule FAK inhibitor that directly inhibits the essential Y397 autophosphorylation and thus, subsequent total phosphorylation and activation of FAK, with an IC50 of 1 μM [49]. FAK14 has high specificity because it did not inhibit nine other recombinant kinases, importantly the Pyk2 homologue of FAK, as well as c-RAF, c-Src, EGFR, VEGFR-3, IGF-1, Met, PDGFR-α, and PI3K, in an in vitro kinase assay [49]. FAK phosphorylation is greatly decreased already at 1 μM in cultured cells [50]. FAK14 dose-dependently reduced mitochondrial function, affecting ATP production, maximum reserve capacity and maximum respiratory capacity of bEnd5 cells within 4 h (Fig. 2a, b). At 10 μM, FAK14 completely abolished mitochondrial bioenergetic function. FAK14 did not affect the number of cells or protein content when cells were incubated for 4 h in Seahorse microwells (without performing subsequent bioenergetics measurements; Fig. 2c, d). SiRNA knockdown of FAK (siFAK) in bEnd5 cells over a 5 day period (to account for the >20 h half-life of FAK [46], and confirmed by westerns for FAK, not shown) also led to mitochondrial dysfunction (Fig. 2e). The effect was not as large as seen with FAK14, perhaps because siRNA mediated knockdown of FAK reduced protein expression by ~80% over the course of 5 days, likely giving cells time to compensate to some extent as well as retaining a small amount of functional FAK. FAK14, as a small molecule inhibitor, would block all molecules with more or less immediate effect.

### S727-STAT3 inhibition causes mitochondrial dysfunction

**Fig. 3:**
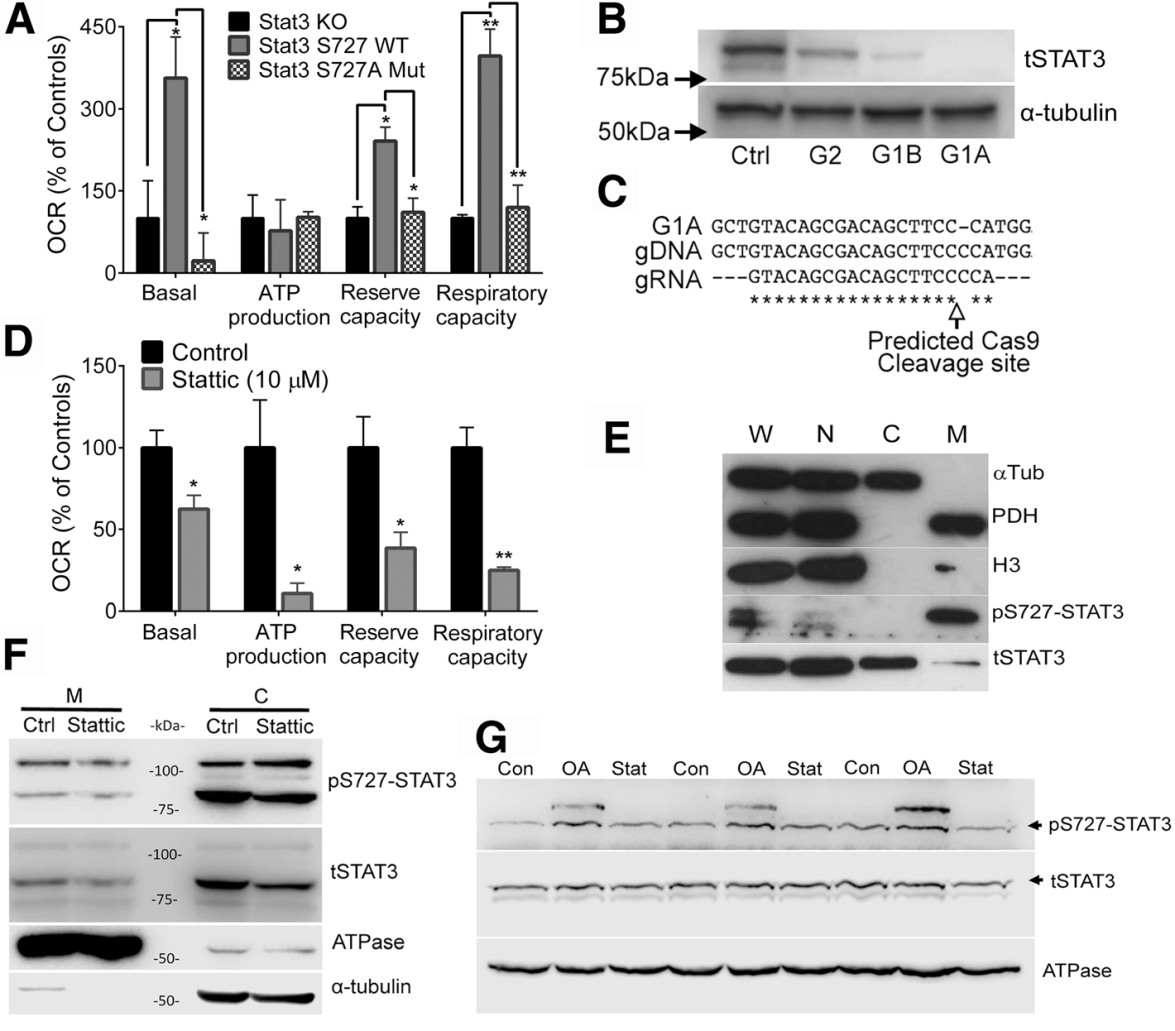
S727-STAT3 inhibition causes mitochondrial dysfunction. **a** Expression plasmids for wildtype (WT), but not functionally dead S727A mutant, STAT3 increased bioenergetics function in bEnd5 cells rendered STAT3−/− by a CRISPR-Cas9 method. *N* = 3. **b** Deletion of STAT3 protein was confirmed by western blot most clearly in cells transfected with the G1A guide RNA (G1A and G1B were replicate conditions of the same Cas9-guideRNA plasmid) which was used for the bioenergetics experiment in (A). **c** DNA sequence data confirmed a point mutation and frame shift in exon 1. **d** Pharmacological inhibition of STAT3 with stattic for 2 h in bEnd5 cells reduced mitochondrial bioenergetic measures. *n* = 3. **e** pS727-STAT3 is predominantly present in the mitochondria and nucleus as shown in Western blots of protein extracts from sub-cellular fractions. Markers = H3 protein, nucleus (N), α-tubulin, cytoplasm (C), PDH, mitochondria (M). W = whole cell lysate. tSTAT3 = total STAT3. **f** Cells treated for 4 h with the STAT3 inhibitor, stattic (10 μM), had less pS727-STAT3 in their isolated mitochondria. The blot is representative of three experiments. **g** pS727-STAT3 was increased in isolated bEnd5 mitochondria treated with okadaic acid (OA: 1 μM) in respiration buffer, as detected by western blotting (3 independent experiments are shown). Stattic did not have any effect. A higher molecular weight band appeared after OA treatment in the pS727-STAT3 blot. A faint band could be seen in the same position after longer exposure of the total STAT3 blot (not shown)

STAT3 was deleted in bEnd5 cells using the CRISPR-Cas9 method. Transfection of these cells with WT plasmids increased bioenergetic function whereas transfection with S727A mutants was without any effect (Fig. 3a). STAT3 protein deletion was confirmed by western blot (Fig. 3b) and the point mutation by sequencing (Fig. 3c). This shows that S727-STAT3 regulates mitochondrial function and is consistent with findings of others [24].

Stattic (6-nitrobenzo[*b*]thiophene 1,1-dioxide, MW = 211) is a small molecule inhibitor of STAT3, selectively blocking STAT3 SH2 domains at an IC50 of 5 μM, inhibiting STAT3 activation, dimerization and nuclear translocation [51]. It has high specificity against STAT3 compared to STAT1, and other transcription factors (c-Myc, Max, Jun) and leads to reduced Y705 and S727 STAT3 (but not JAK1, JAK2, c-Src, AKT, JNK or ERK) phosphorylation [29, 51, 52]. Stattic caused mitochondrial dysfunction in bEnd5 cells as early as 2 h (Fig. 3d). Most of the pS727-STAT3 was present in the nucleus and mitochondria, as shown by sub-cellular fractionation (Fig. 3e), consistent with other studies [24]. Stattic treatment of bEnd5 cells caused a reduction in mitochondrial pS727-STAT3 within 4 h (Fig. 3f).

To identify where S727-STAT3 is phosphorylated and dephosphorylated, freshly isolated bEND5 mitochondria were treated for 2 h with stattic or the serine/threonine phosphatase inhibitor, okadaic acid. Stattic did not have an effect, suggesting that STAT3 is phosphorylated in the cytoplasm before being imported, as others have found [53]. Okadaic acid increased pS727-STAT3 (Fig. 3g). A higher molecular weight pS727 band was visible after treatment with okadaic acid in a position where a faint band of total STAT3 could be seen after longer exposure (not shown).

### FAK inhibition reduces mitochondrial S727-STAT3

**Fig. 4:**
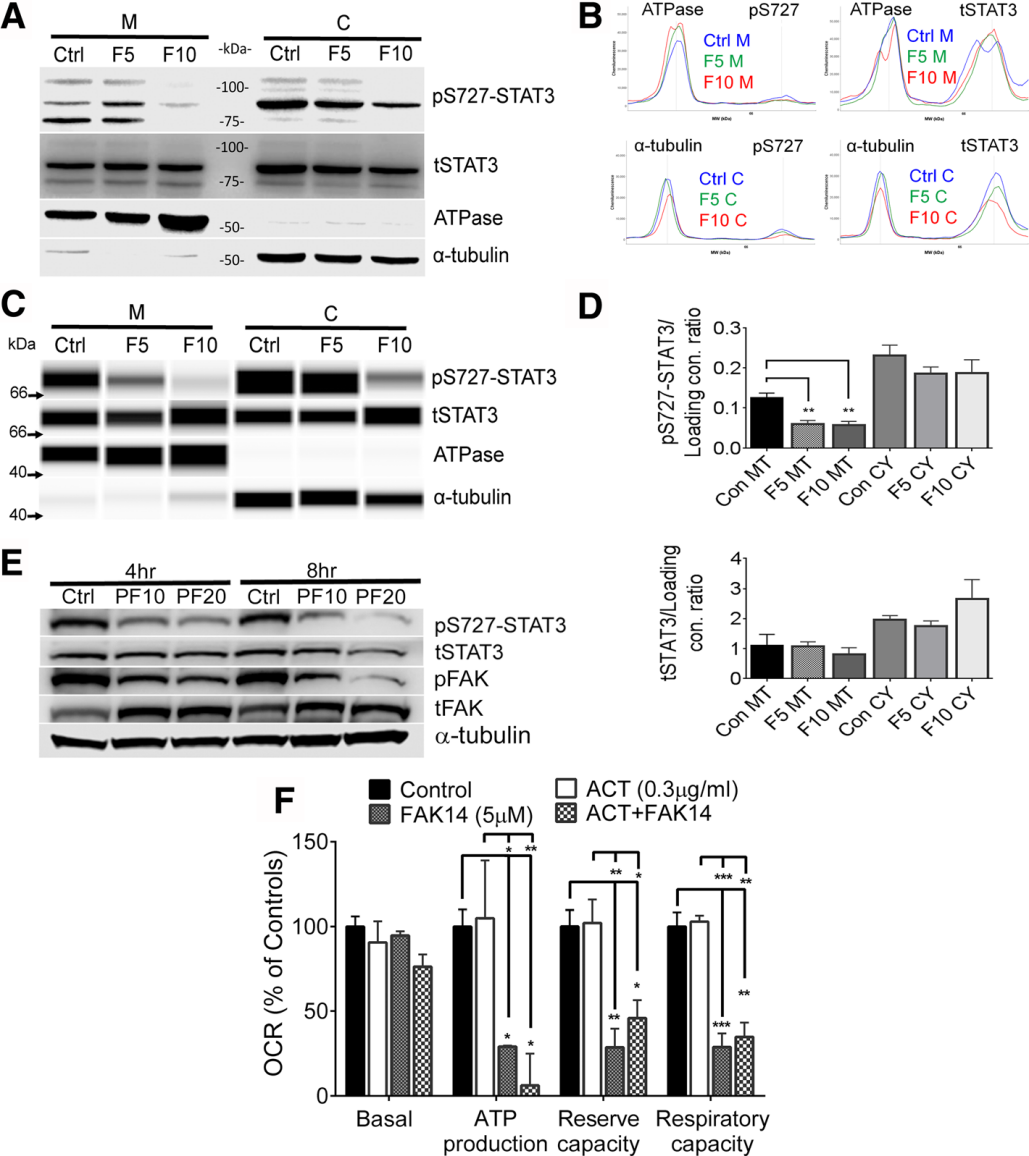
FAK inhibits mitochondrial S727-STAT3 phosphorylation. **a** A 4 h FAK14 treatment of bEnd5 cells reduced pS727-STAT3 in both the mitochondrial and cytoplasmic fractions. Blots are representative for 5 experiments. **b** This reduction was confirmed by quantitative capillary western blotting with representative chemiluminescent spectrograms and synthetic bands (**c**). **d** Quantitation was performed of spectrograms confirmed a clear and significant decrease in pS727-STAT3 following 4 h FAK14 treatment in the mitochondrial fractions (*n* = 3). **e** Treatment with another more lipophilic FAK antagonist (PF573228: PF at 10 or 20 μM) for 4 or 8 h showed decreases in pS727-STAT3 in conjunction with decreased pFAK in whole cell lysates. **f** Incubation with the global transcriptional inhibitor actinomycin D (0.3 μg/ml, 4 h) did not significantly change mitochondrial bioenergetics under control or FAK14 conditions

FAK inhibitor FAK14 (Fig. 4a) caused a reduction in mitochondrial pS727-STAT3 after 4 h. The higher molecular weight pS727-STAT3 could be detected in the freshly isolated mitochondria (Fig. 4a) and disappeared upon FAK inhibition with FAK14. The results were confirmed by a quantitative capillary western method, as shown in representative traces (Fig. 4b), which were converted with software to synthetic bands for presentation purposes (Fig. 4c), and quantified (Fig. 4d). Another FAK inhibitor, PF573228, also reduced pS727-STAT3, in concert with pFAK, as shown in whole cell lysates (Fig. 4e). Treatment with the transcription inhibitor, actinomycin, for 4 h did not significantly affect mitochondrial bioenergetics, nor the effects of FAK inhibition (Fig. 4f), consistent with the known non-transcriptional role of pS727-STAT3 [23]. The results so far suggest that integrin signaling through FAK promotes mitochondrial function by activating STAT3 on S727.

### S727-STAT3 activators rescue mitochondrial function and cells against FAK inhibition

**Fig. 5:**
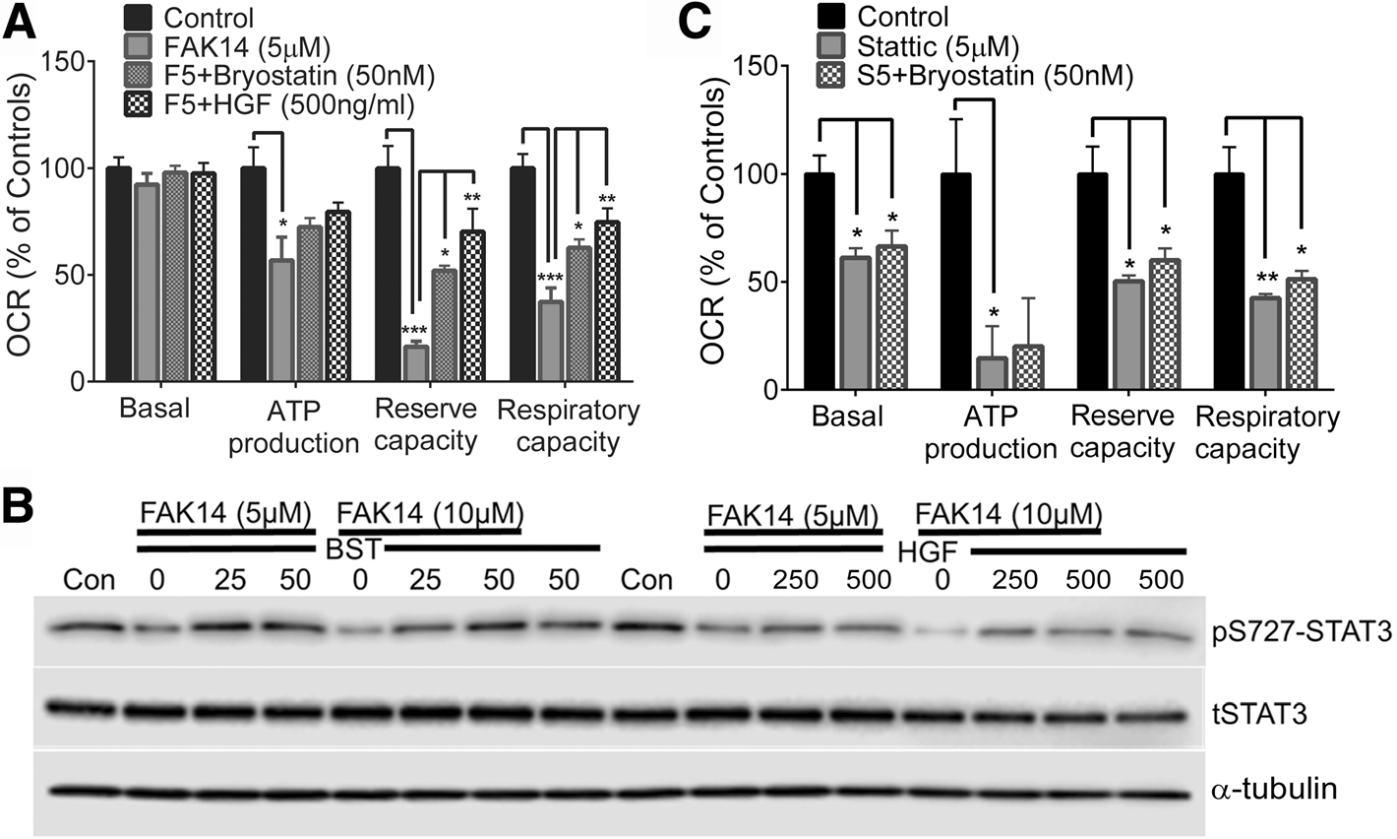
pS727-STAT3 stimulation preserves mitochondrial bioenergetics against FAK inhibition. **a** Bryostatin and HGF preserved reserve and respiratory capacity in bEnd5 cells incubated with FAK14 (5 μM, F5) for 4 h. Data are mean ± SEM from 3 independent experiments. *p < 0.05, **p < 0.01, **p < 0.001. **b** Western blots of whole cell lysates showing that bryostatin (BST) and HGF preserve pS727-STAT3 in the presence of FAK14 (representative for 3 independent experiments). **c** Bryostatin does not affect the mitochondrial bioenergetics dysfunction caused by STAT3 inhibition with stattic. *N* = 3

**Fig. 6:**
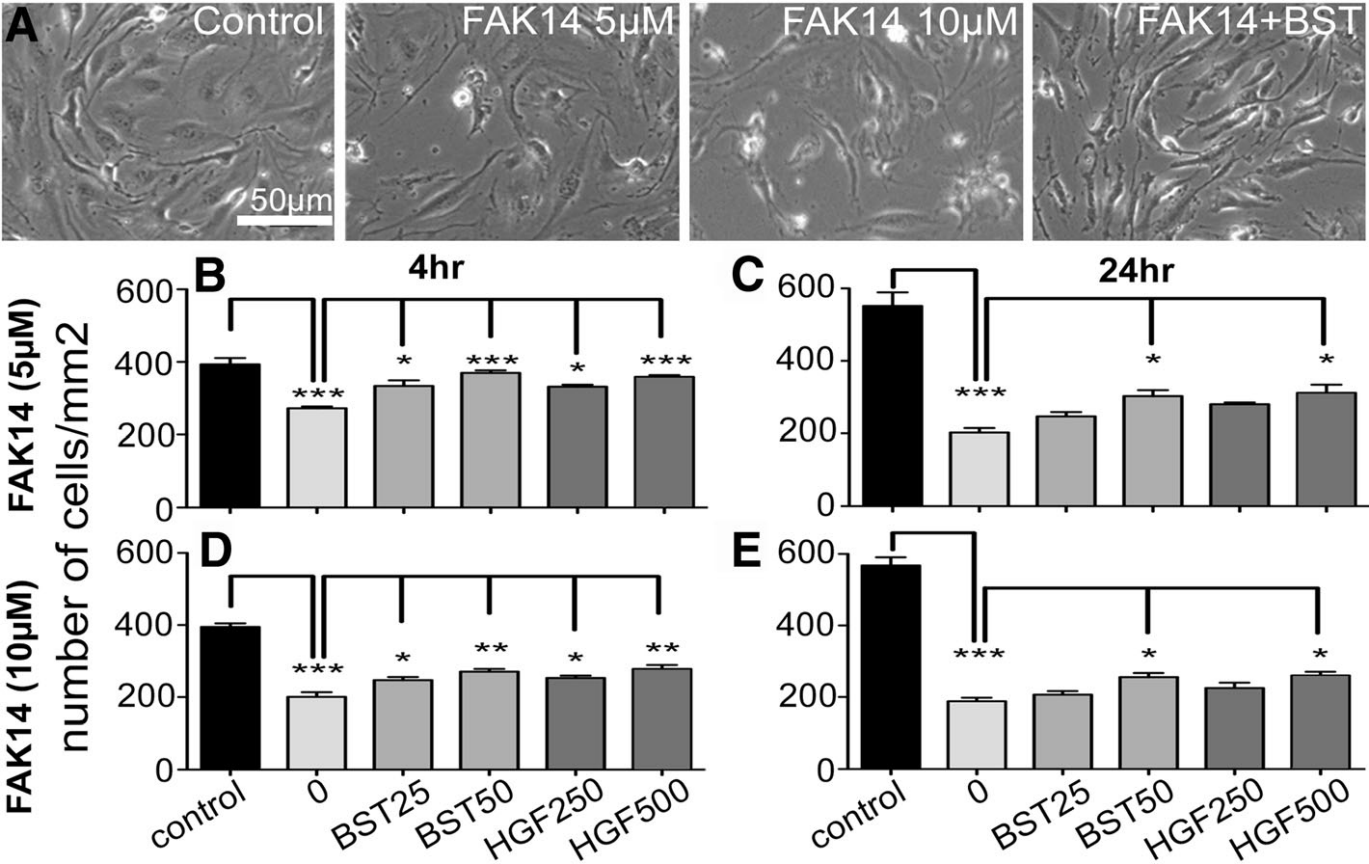
Bryostatin and HGF reduce bEND5 cell death due to FAK inhibition. **a** Images are representative of bEnd5 cells grown for 4 h with 5 μM FAK14 +/−bryostatin (BST) in regular tissue culture wells. **b** to **e**) Cell counts show that bryostatin and HGF can promote survival despite FAK inhibition with 5 or 10 μM FAK14 for 4 or 24 h. Data are mean ± SEM from 3 independent experiments. *p < 0.05, **p < 0.01 and ***p < 0.001

Bryostatin-1 and HGF increase phosphorylation of S727-STAT3 via PKC, and ERK activation, respectively [34, 54]. Here, both mitigated the reduced mitochondrial maximum reserve and respiratory capacity caused by a 4 h FAK14 treatment of bEnd5 cells (Fig. 5a). Further, FAK14 dose-dependently decreased pS727-STAT3, which was prevented by bryostatin and HGF (Fig. 5b). The substantial changes in pS727-STAT3 are likely not resulting from changes in cell survival (Fig. 6) because the total STAT3 and actin loading controls were similar. Bryostatin could not reduce mitochondrial dysfunction caused by stattic (Fig. 5c), evidence that its protective effects against FAK inhibition (Fig. 5a, and below) were mediated by STAT3 activation.

BEnd5 cell survival was assessed in regular tissue culture plates. FAK14 dose- and time-dependently caused loss of bEnd5 cells (Fig. 6a-e), up to more than 50% (Fig. 6d, e), and may have reduced proliferation between 4 and 24 h (controls in Fig. 6b vs. c and d vs. e). No cell loss was observed in the bioenergetics experiments (Fig. 2), probably due to the different culture conditions. Bryostatin and HGF promoted bEnd5 cell survival caused by FAK14 treatment at both 4 h (Fig. 6a, b, d) and 24 h (Fig. 6c, e).

### FAK inhibition causes mitochondrial depolarization and ROS production

**Fig. 7:**
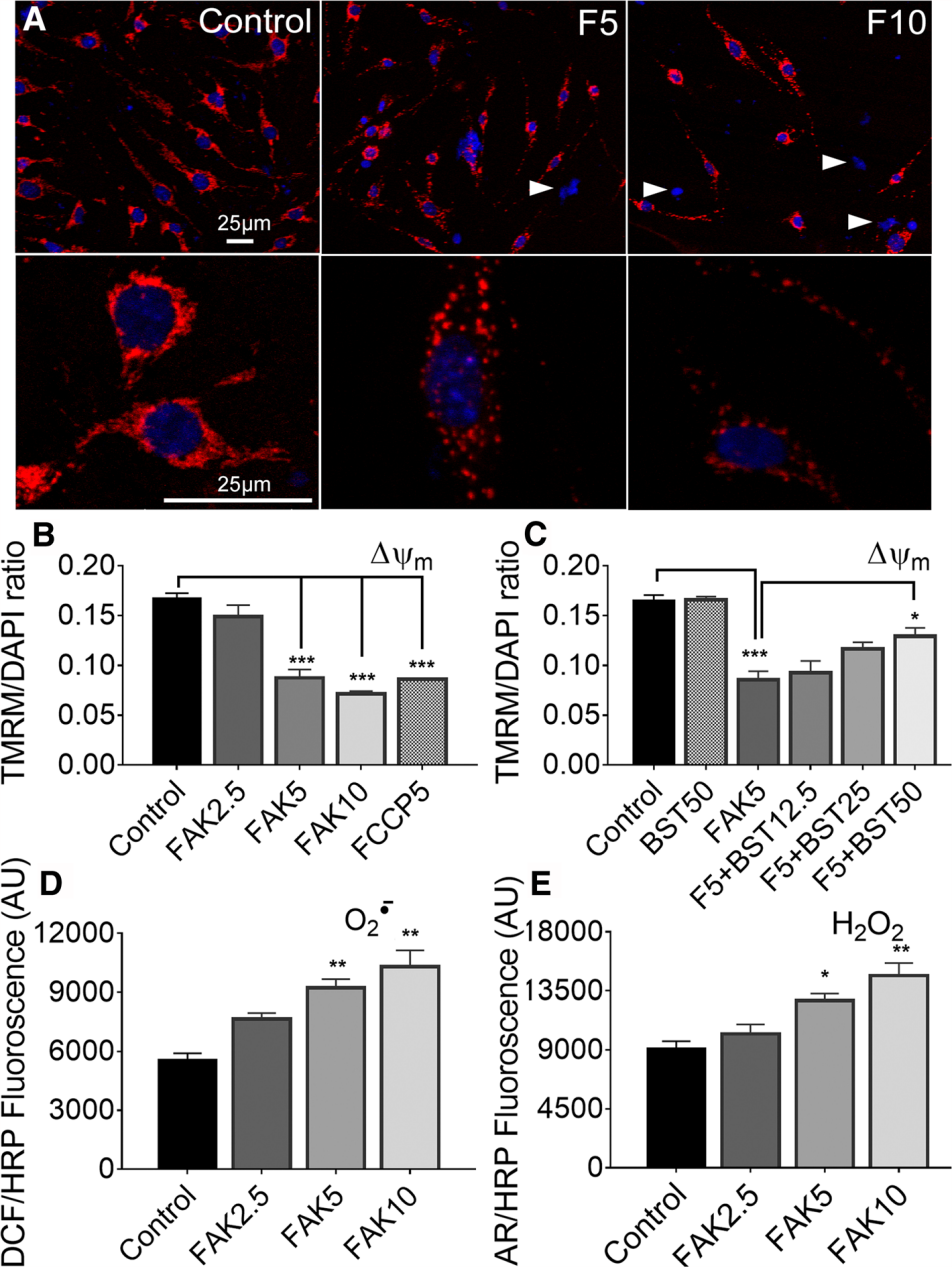
FAK inhibition depresses mitochondrial membrane potential and increases ROS production. **a** FAK inhibition with FAK14 for 4 h caused a dose-dependent reduction in mitochondrial membrane potential, as shown by confocal images of red TMRM fluorescence in bEnd5 cells. Cells with complete mitochondrial failure are indicated by arrowheads and detectable only by blue nuclear Hoechst staining. **b** Spectrometry of TMRM in extracts was used to quantify the effects, and shows that 10 μM FAK14 (FAK10) reduced the membrane potential to the same level as FCCP. Values were normalized to nuclear DAPI staining to account for differences in cell numbers. Data are mean ± SEM from 3 independent experiments. *p < 0.05, **p < 0.01, ***p < 0.001. **c** Bryostatin (BST) dose-dependently preserved mitochondrial membrane potential in the presence of FAK14. *N* = 3. FAK14 dose-dependently induced formation of **d**) superoxide (O_2_^.-^) and **e**) H_2_O_2_. *N* = 3

A normal mitochondrial membrane potential (ΔΨ_m_) is essential for ATP generation [55]. FAK14 caused a dose-dependent reduction in TMRM fluorescent signal, a measure of ΔΨm, in bEnd5 cells after 4 h as shown by confocal images (Fig. 7a) and spectrofluorometry of cell extracts (Fig. 7b). At 10 μM, FAK14 caused a complete loss of membrane potential to levels seen with the FCCP uncoupler of mitochondrial oxidative phosphorylation. Bryostatin increased mitochondrial polarization, as assessed with TMRM, in a dose-dependent manner in the presence of FAK14, but did not have an effect by itself (Fig. 7c). Mitochondrial failure can be induced by excessive ROS formation [56]. A 4 h treatment with FAK14 dose-dependently increased superoxide (Fig. 7d) and H_2_O_2_ (Fig. 7e) formation. Bryostatin did not affect this FAK14-induced ROS formation (data not shown).

## Discussion

The present study demonstrates that ECM–integrin-FAK signaling regulates mitochondrial bioenergetics via pS727-STAT3. This adds an important component to what was known about integrin signaling functions. ECM-integrin binding is important for cell adhesion and regulates actin organization, cell movement and cell cycle control [57]. Integrins can play an important role in cell survival, especially of cells that are attached to basement membranes, such as endothelial cells which undergo anoikis when attachment is disrupted [19]. Disrupted integrin signaling can induce apoptosis by activating the pro-apoptotic Bad and Bax or reducing anti-apoptotic Bcl-2 [3, 58]. RGDS peptide can trigger pro-apoptotic induction and activation of caspase 8 and 9 in human endothelial cells [59]. The P11 antagonist of αvβ3 integrin can cause apoptosis of human umbilical vein endothelial cells by up-regulating p53 expression resulting in caspase activation [60]. Moreover, lengthy low bioenergetics leads to pathological mitochondrial pore formation and cytochrome-C release thus initiating apoptotic death [17, 18, 61]. Our data suggest that the integrin-FAK-STAT3 pathway is involved in suppressing such cell death mechanisms by maintaining mitochondrial function and integrity. This is consistent with the finding that fibronectin knockdown induces apoptosis in rat mesangial cells in a mitochondria-dependent manner, mainly as a result of cytochrome c release and downstream caspase-3 and −9 activation [62]. Conversely, laminin and fibronectin protect pancreatic cancer cells from death by mechanisms involving the inhibition of both mitochondrial depolarization and caspase activity [63]. Previously, we have shown that an αvβ3 agonist peptide can rescue endothelial cells after contusive spinal cord injury in adult mice [64].

Integrin-FAK signaling also can regulate mitochondrial biogenesis and morphology. FAK inactivation in cardiomyocytes causes structural abnormalities in mitochondria [65]. Moreover, FAK interacts with mitochondrial transcriptional cascades and enhances mechanical stress-induced mitochondrial biogenesis [66]. It remains to be determined how the FAK-STAT3 pathway affects biogenesis and morphology.

Most of our experiments were conducted with VTN as an integrin ligand. Its effects on mitochondrial function were mediated at least by αvβ3 integrin as shown by the inhibitory effects of the P11 peptide. Fibronectin and laminin also had a stimulating effect, consistent with their role in promoting endothelial cell survival [3, 67, 68], whereas RGD inhibition reduced bioenergetic function. Other ECM molecules also utilize RGD integrins and it remains to be determined whether additional basement membrane or ECM molecules [69] are involved in regulating mitochondrial function. Vitronectin is present in the basement membrane but also at high concentrations in the blood [70, 71]. It will be interesting to determine the role of plasma VTN on mitochondrial function or whether integrin activation is predominantly ab-luminal, luminal, or both in the vasculature. FAK may be predominantly clustered with the VTN receptor on the ab-luminal side of endothelial cells, where they would make contact with the basement membrane [72]. If so, it is possible that pathological VTN leakage from the blood would promote survival of endothelial cells.

Our data suggest that FAK activates S727-STAT3. It remains to be determined which pathways downstream of FAK might be involved, but could include the serine kinases JNK1 [22] or ERK [73]. The latter is also activated by HGF [34]. The bryostatin results suggest that one or more of the PKC kinases can activate STAT3 even under reduced FAK signaling conditions. FAK can also be activated by growth factor receptors and G-protein-linked receptors [74, 75] raising the possibility that they too could promote to mitochondrial function. The ECM may be involved in orchestrating these miscellaneous extracellular regulators. Our acute pharmacological approach with FAK14 and stattic showed a remarkably complete cessation of mitochondrial bioenergetic function within 4 h. A mitochondrial role for STAT3 has been convincingly demonstrated with STAT3 S727A mutants suppressing bioenergetic function by ~50-70% [24]. Our results, validate these findings by confirming that expression of STAT3 S727A mutants cannot increase bioenergetic function in STAT3 knockout cells using CRISPR-Cas9, whereas wild type STAT3 controls can. Other pathways that regulate STAT3 pS727 activation might also be involved under more physiological conditions. For example, CNTF can promote mitochondrial function through NFKB expression and activity in dorsal root ganglion neurons [76]. It remains to be determined whether this effect is fully transcription-dependent or might also involve S727-STAT3 activation.

The mechanisms and roles of STAT3 within the mitochondria are not well-understood. Our results with actinomycin are consistent with the known non-transcriptional nature of the role of STAT3 in mitochondrial function [24, 26], and support a non-transcriptional effect of FAK inhibition on mitochondria. In an apparent contradiction, in keratinocytes, S727-STAT3 can repress transcription of mitochondrial ETC genes, but no functional measures were reported [77]. Our finding that pS727-STAT3 is mainly found in the mitochondrial and nuclear fractions, but less in the cytoplasm, is consistent with the findings that STAT3 translocates upon S727 phosphorylation in the cytoplasm [53]. Moreover, treatment of isolated mitochondria with stattic, known to reduce phosphorylation and translocation [51], did not reduce mitochondrial pS727-STAT3. This suggests that the effects of integrin or FAK inhibition are mediated by a reduced translocation to the mitochondria, which may be dependent on chaperones such as HSPs [78, 79]. We also find for the first time that dephosphorylation of S727-STAT3 occurs within the mitochondria, as isolated mitochondria treated for 2 h with the serine/threonine phosphatase inhibitor, okadaic acid, had more pS727-STAT3. The rapidity suggests that mitochondrial pS727-STAT3 is short-lived and its constant import dependent on external stimuli which activate FAK signaling. This would represent a mechanism for rapid adjustments to cellular energy demands. Okadaic acid is known to inhibit PP2A (and PP1) which has been associated with pS727-STAT3 dephosphorylation in the cytoplasm [80]. PP2A has been found in mitochondria [81] and has been linked to mitochondrial bioenergetics by promoting mitochondrial division through Drp1 dephosphorylation [82].

We detected an ~100 kDa pS727-STAT3 in okadaic acid-treated or freshly isolated mitochondria. This form may also have been found in avian neurons upon CNTF stimulation, showing a similar short-lived nature and dependency on persistent signaling [83]. A higher molecular weight STAT3 can also be detected in Cos1 cells (Fig. 4 in [23] and splenocytes (Fig. 3 in [84]). Our results suggest that only a very minor fraction of mitochondrial STAT3 exists in this form and might be newly imported STAT3 which is post-translationally modified.

Mitochondrial STAT3 is thought to maintain optimal oxidative phosphorylation, by interacting with ETC complexes I, II, and V, and inhibiting formation of the mitochondrial permeability transition pore which would lead to loss of mitochondrial membrane potential [26, 29]. Our results suggest that ligand-dependent integrin-FAK activation of S727-STAT3 is an important mechanism which maintains a physiological mitochondrial function. Together, our data support the idea that the integrin-FAK signaling pathway, through STAT3, prevents the loss of mitochondrial membrane potential and decreased ATP synthesis, which are key mediators of mitochondrial dysfunction and subsequent triggers for release of pro-apoptotic factors and cell death [85].

Our results also suggest that this pathway promotes the potential antioxidant role of STAT3. In this scenario, STAT3 binds to complex I iron sulfur clusters with its cysteines being oxidized to serve as electron donors, thus reducing ROS generation [30, 31]. Indeed, STAT3 can reduce ROS formation in cardiomyocytes [30], preventing excessive ROS levels which can lead to cell death [56]. Integrins and ECM molecules have been found to reduce [86, 87] or promote [88, 89] ROS production. It will be interesting to test whether pS727-STAT3 is differentially regulated under those conditions or in those cells. Mitochondrial ROS are also involved in physiological processes, including proliferation, differentiation and migration [90]. It remains to be determined whether the integrin-STAT3 pathway plays a role in these physiological functions. Our finding that bryostatin did not reduce superoxide and H_2_O_2_ formation caused by FAK inhibition may explain why it was not fully effective in rescuing mitochondrial function or cell survival, despite restoring normal pS727-STAT3 levels. This raises the possibility that FAK can regulate mitochondrial function through other pathways.

Our study also explored a potential therapeutic strategy for endothelial cells with dysfunctional integrin signaling such as is seen after traumatic detachment, by circumventing integrin-FAK signaling through direct STAT3 activation. Our results are proof of principle that this can be achieved, also because we have seen similar decreases in bioenergetics, pS727-STAT3, and mitochondrial membrane potential after FAK inhibitor treatment in primary endothelial cells derived from adult mouse brain (Visavadiya et al., unpublished results). However, although it remains to be seen whether this therapeutic approach can be optimized and applied to animal models of disease. This is a reasonable expectation because bryostatin is being explored as a treatment for Alzheimer’s disease [37] and HGF is in clinical trials for spinal cord injury [38]. This new therapeutic strategy could have implications for disorders where endothelial cells are disturbed, e.g., to maintain proper BBB integrity during neurological or other insults. FAK inhibitors are in clinical trials for solid tumors [91, 92] due to their inhibition of angiogenesis and cell adhesion [49, 93]. Our results suggest that mitochondrial dysfunction caused by such agents could also explain some of the effects. In apparent contrast to our proposed effects of FAK inhibition on bioenergetics function and ROS, mitochondrial ROS could trigger gastric cancer progression by stimulating cancer cell migration via the β5-integrin induction [94]. Also, mitochondrial bioenergetic dysfunction caused by mitochondrial DNA mutations leads to increased motility and migration in cytoplasmic hybrid cells “cybrid cells” which harbor A3243T mutation in the leucine transfer RNA gene through a mechanism involving ECM molecules [95]. It remains to be determined whether the integrin-FAK-STAT3 pathway plays different roles in different cell types, and whether it is absent in cancer cells which defeat anoikis to metastasize.

## Conclusions

In conclusion, our data suggest that integrin-FAK signaling maintains mitochondrial bioenergetic function by maintaining membrane potential and ETC function, thus reducing ROS formation, via pS727-STAT3. Further, we propose a treatment strategy that can circumvent dysfunction of integrin signaling which may help to develop new neuroprotective treatments. Our finding also provides a platform to investigate fundamental mechanisms of how ligand binding to integrins modulate physiological mitochondrial bioenergetic function in cells.

## Abbreviations

ACT: Actinomycin D
AKT: v-Akt murine thymoma viral oncogene/protein kinase B (PKB)
ANOVA: One way analysis of variance
ATP: Adenosine triphosphate
ATPase: Adenosine triphosphate synthase
BBB: Blood–brain barrier
Bcl-2: B-cell lymphoma 2
bEnd5: Mouse brain endothelioma cell line
bit1: Bcl-2 inhibitor of transcription
BST: Bryostatin
Cas9: CRISPR associated protein 9
CNS: Central nervous system
CNTF: Ciliary neurotrophic factor
CRISPR: Clustered regularly interspaced short palindromic repeats
DAPI: 4′,6-diamidino-2-phenylindole
DCFH_2_-DA: 2-,7-dichlorodihydrofluorescein diacetate
DMEM: Dulbecco’s modified eagle medium
DNA: Deoxyribonucleic acid
ECL: Enhanced chemiluminescence
ECM: Extracellular matrix
EGTA: Ethylene glycol-bis(β-aminoethyl ether)-N,N,N’,N’-tetraacetic acid
ERK: Extracellular signal-regulated kinases
ETC: Electron transport chain
F10: FAK14 (10 μM)
F5: FAK14 (5 μM)
FAK: Focal adhesion kinase
FAK14: 1,2,4,5-Benzenetetramine tetrahydrochloride
FCCP: Carbonyl cyanide 4-(trifluoromethoxy) phenylhydrazone
FN: Fibronectin
H_2_O_2_: Hydrogen peroxide
H3: Histone H3
HEPES: 4-(2-hydroxyethyl)-1-piperazineethanesulfonic acid
HGF: Hepatocyte growth factor
HRP: Horseradish peroxidase
HSPs: Heat shock proteins
JNK: c-Jun N-terminal kinases
KCl: Potassium chloride
KH_2_PO_4_: Potassium phosphate monobasic
KOH: Potassium hydroxide
LAM: Laminin
MgCl_2_: Magnesium chloride
NFKB: Nuclear factor kappa B
O_2_^.-^: Superoxide
OA: Okadaic acid
OCR: Oxygen consumption rate
PDH: Pyruvate dehydrogenase
PF10: PF573228 (10 μM)
PF20: PF573228 (20 μM)
PF573228: 3,4-Dihydro-6-[[4-[[[3-(methylsulfonyl)phenyl]methyl]amino]-5-(trifluoromethyl)-2-pyrimidinyl]amino]-2(1*H*)-quinolinon
PKC: Protein kinase C
pS727-STAT3: Phosphorylated serine 727 residue of STAT3
PVDF: Polyvinylidene difluoride
pY397-FAK: Phosphorylated tyrosine 397 residue of FAK
RGD: Arg-Gly-Asp
RGDS: Arg-Gly-Asp-Ser
RIPA: Radioimmunoprecipitation assay
RNA: Ribonucleic acid
ROS: Reactive oxygen species
S5: Stattic (5 μM)
S727-STAT3: Serine 727 residue of STAT3
SDS-PAGE: Sodium dodecyl sulfate-polyacrylamide gel electrophoresis
siFAK: Small interfering RNA for FAK
siRNA: Small interfering RNA
STAT3: Signal transducer and activator of transcription 3
TBST: Tris-buffered saline tween 20
TMRM: Tetramethylrhodamine, methyl ester
tSTAT3: Total signal transducer and activator of transcription 3
VTN: Vitronectin
α*v*β*3*: alpha v beta 3
ΔΨ_m_: Membrane potential
