# Erratum to: Integrin-FAK signaling rapidly and potently promotes mitochondrial function through STAT3

**DOI:** 10.1186/s12964-017-0167-0

**Published:** 2017-03-27

**Authors:** Nishant P. Visavadiya, Matthew P. Keasey, Vladislav Razskazovskiy, Kalpita Banerjee, Cuihong Jia, Chiharu Lovins, Gary L. Wright, Theo Hagg

**Affiliations:** Department of Biomedical Sciences, Quillen College of Medicine, East Tennessee State University, Building 178, Maple Ave, PO Box 70582, Johnson City, TN37614 USA

## Erratum

Unfortunately, after publication of this article [1], it was noticed that the Acknowledgements and Funding sections were incomplete. The Acknowledgements section currently reads, “We are grateful for the technical support by Aruna Visavadiya, Ying Li, and Rhesa Dykes” and the Funding section currently reads, “This work was supported by NIH grant NS45734 and ETSU medical school funds”. The full, corrected sections can be seen below.

**Acknowledgements**

We are grateful for the technical support by Aruna Visavadiya, Ying Li, and Rhesa Dykes. Dr. Britta Engelhardt (Theodor Kocher institute) is thanked for providing the bEnd5 cells.

**Funding**

This work was supported by NIH grant NS45734 and in part by NIH grant C06RR0306551 and the ETSU College of Medicine.
