# Correction to: Integrin-FAK signaling rapidly and potently promotes mitochondrial function through STAT3

**DOI:** 10.1186/s12964-020-00577-y

**Published:** 2020-04-20

**Authors:** Nishant P. Visavadiya, Matthew P. Keasey, Vladislav Razskazovskiy, Kalpita Banerjee, Cuihong Jia, Chiharu Lovins, Gary L. Wright, Theo Hagg

**Affiliations:** Department of Biomedical Sciences, Quillen College of Medicine, East Tennessee State University, Building 178, Maple Ave, PO Box 70582, Johnson City, TN37614 USA

**Correction to: Cell Commun Signal (2016) 14:32**

**https://doi.org/10.1186/s12964-016-0157-7**

Unfortunately, after publication of this article [1], it was noticed that the Acknowledgements and Funding sections were incomplete. The Acknowledgements section currently reads, “We are grateful for the technical support by Aruna Visavadiya, Ying Li, and Rhesa Dykes” and the Funding section currently reads, “This work was supported by NIH grant NS45734 and ETSU medical school funds”. The full, corrected sections can be seen below.

**Acknowledgements**

We are grateful for the technical support by Aruna Visavadiya, Ying Li, and Rhesa Dykes. Dr. Britta Engelhardt (Theodor Kocher institute) is thanked for providing the bEnd5 cells.

**Funding**

This work was supported by NIH grant NS45734 and in part by NIH grant C06RR0306551 and the ETSU College of Medicine.

Further to this, a duplicate image in Fig. 4e was reported. The correct image is presented in this correction article.

**Fig. 4:**
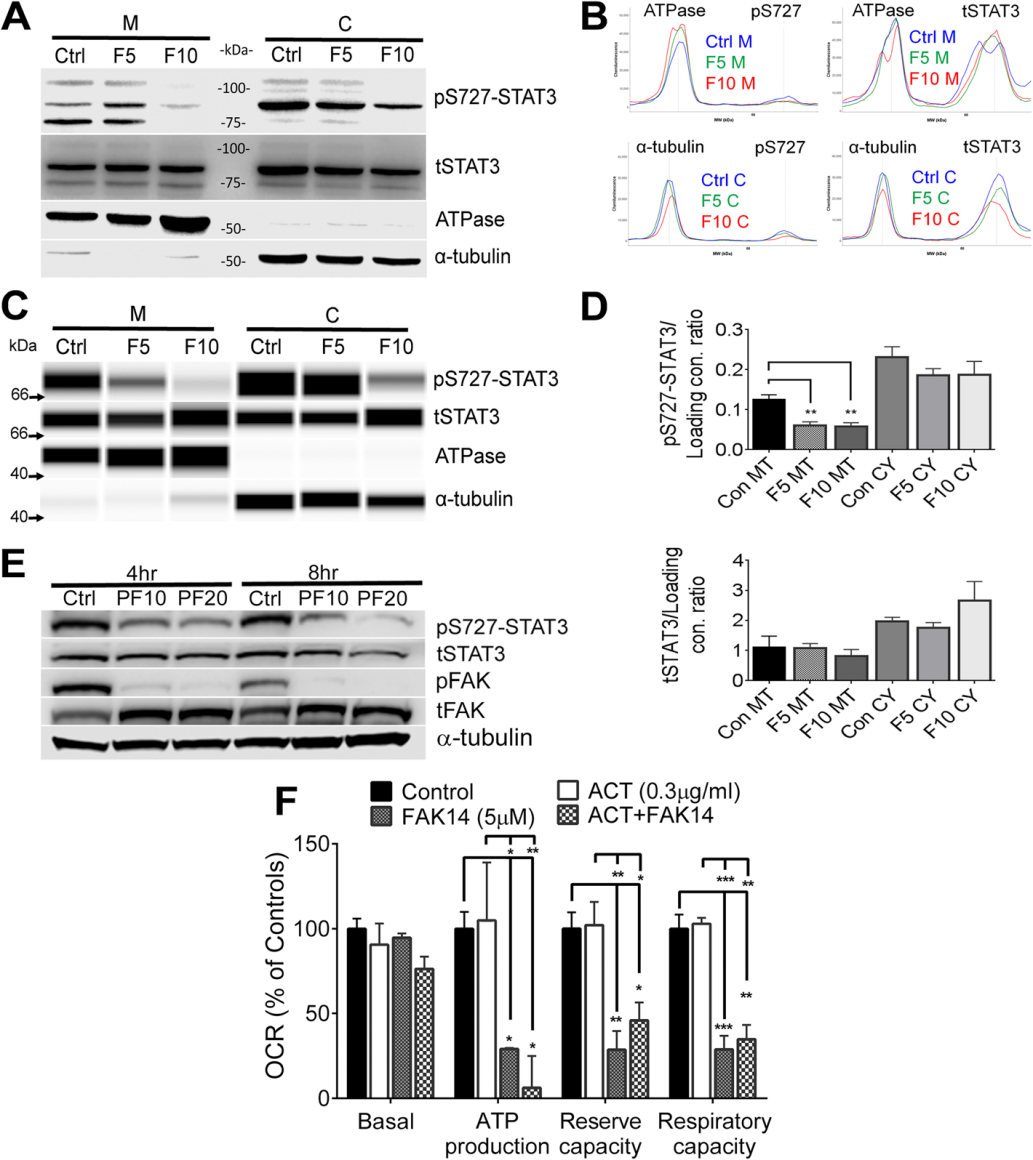
FAK inhibits mitochondrial S727-STAT3 phosphorylation. **a** A 4 h FAK14 treatment of bEnd5 cells reduced pS727-STAT3 in both the mitochondrial and cytoplasmic fractions. Blots are representative for 5 experiments. **b** This reduction was confirmed by quantitative capillary western blotting with representative chemiluminescent spectrograms and synthetic bands (**c**). **d** Quantitation was performed of spectrograms confirmed a clear and significant decrease in pS727-STAT3 following 4 h FAK14 treatment in the mitochondrial fractions (*n* = 3). **e** Treatment with another more lipophilic FAK antagonist (PF573228: PF at 10 or 20 μM) for 4 or 8 h showed decreases in pS727-STAT3 in conjunction with decreased pFAK in whole cell lysates. **f** Incubation with the global transcriptional inhibitor actinomycin D (0.3 μg/ml, 4 h) did not significantly change mitochondrial bioenergetics under control or FAK14 conditions
